# Predicting clinical outcomes among hospitalized COVID-19 patients using both local and published models

**DOI:** 10.1186/s12911-021-01576-w

**Published:** 2021-07-24

**Authors:** William Galanter, Jorge Mario Rodríguez-Fernández, Kevin Chow, Samuel Harford, Karl M. Kochendorfer, Maryam Pishgar, Julian Theis, John Zulueta, Houshang Darabi

**Affiliations:** 1 Departments of Medicine and Pharmacy Systems, Outcomes and Policy, University of Illinois At Chicago (UIC), Chicago, USA; 2 Department of Neurology, Clinical Informatics Fellowship, UIC, Chicago, USA; 3 College of Medicine, UIC, Chicago, USA; 4 Department of Mechanical and Industrial Engineering, UIC, Chicago, USA; 5 Department of Family and Community Medicine, UIC, Chicago, USA; 6 Department of Psychiatry, UIC, Chicago, USA

**Keywords:** Mortality, Hospitalization, COVID-19, Statistical model, Prediction, Model generalizability

## Abstract

**Background:**

Many models are published which predict outcomes in hospitalized COVID-19 patients. The generalizability of many is unknown. We evaluated the performance of selected models from the literature and our own models to predict outcomes in patients at our institution.

**Methods:**

We searched the literature for models predicting outcomes in inpatients with COVID-19. We produced models of mortality or criticality (mortality or ICU admission) in a development cohort. We tested external models which provided sufficient information and our models using a test cohort of our most recent patients. The performance of models was compared using the area under the receiver operator curve (AUC).

**Results:**

Our literature review yielded 41 papers. Of those, 8 were found to have sufficient documentation and concordance with features available in our cohort to implement in our test cohort. All models were from Chinese patients. One model predicted criticality and seven mortality. Tested against the test cohort, internal models had an AUC of 0.84 (0.74–0.94) for mortality and 0.83 (0.76–0.90) for criticality. The best external model had an AUC of 0.89 (0.82–0.96) using three variables, another an AUC of 0.84 (0.78–0.91) using ten variables. AUC’s ranged from 0.68 to 0.89. On average, models tested were unable to produce predictions in 27% of patients due to missing lab data.

**Conclusion:**

Despite differences in pandemic timeline, race, and socio-cultural healthcare context some models derived in China performed well. For healthcare organizations considering implementation of an external model, concordance between the features used in the model and features available in their own patients may be important. Analysis of both local and external models should be done to help decide on what prediction method is used to provide clinical decision support to clinicians treating COVID-19 patients as well as what lab tests should be included in order sets.

## Background

The coronavirus disease 2019 (COVID-19) caused by the SARS-CoV-2 has been devastating compared to other viruses (seasonal, avian and swine influenza), in regard to both the morbidity and mortality and its economic impact, despite advancements in medical care since the Spanish Flu of 1918 [1]. COVID-19 has had a dramatic impact on health systems globally and the US economy despite assistance from the US Federal government, via the CARES Act [2] and other funding programs.

The COVID-19 pandemic occurred quickly and was rapidly followed by a massive production of academic output, including prediction models for a variety of clinical outcomes; the initial models for hospital outcomes came from the city of Wuhan in the Hubei province of China, where the initial cases were discovered. From there, models around the globe surged and were likely integrated into many hospital guidelines. However, it is unclear if those models could be applied to local cohorts. Having a rapidly available and accurate prediction model for COVID-19 patients being admitted from the emergency department (ED) would be useful for making accurate triage and prognostic assessments to inform decisions regarding treatment and resource allocation. While knowledge of the likelihood of death in those sent home from the ED would also be of interest, this requires longitudinal data which is often not as readily available. The value of appropriate triage decisions is important, especially in time when resources are stretched.

The growth in the volume of readily available healthcare data has facilitated the development of artificial intelligence-based models; however, a significant factor limiting the utility of dissemination of such models is the issue of generalizability. For example, the earliest computer-aided decision models evaluating abdominal pain were not able to be replicated in different institutions [3]. A mortality prediction tool in acute alcoholic pancreatitis (Ranson’s criteria) [4] developed in a small cohort has a wide acceptance compared to superior scoring tools [5].

One of the most popular predictions tools in clinical use today is the 2013 ACC/AHA Guideline on the Assessment of Cardiovascular Risk [6]. This risk tool uniformly overestimated risk in non-diabetic patients in a large, multi-ethnic, socioeconomically group of patients in California [7].

We performed an analysis of how well published and self-developed models would predict clinical outcomes after admission on a cohort of diverse urban patients in Chicago. Our self-developed models were trained using data from our local patient cohort. Published, external models were not re-trained with our cohort’s data. We aim to close the gap in the understanding if COVID-19 prediction models on mortality and criticality could be potentially used in local cohorts despite ethnic, geographic and timeline differences. We postulate that due to our incomplete understanding of the pathophysiology, ethnic, racial and socioeconomic differences by location, and improving treatment over time, that models may not predict well in a cohort different than their validation and development cohorts.

## Methods

### University of Illinois Hospital (UIH) Cohort

UIH is a tertiary, academic teaching hospital in Chicago. The UIC Institutional Review Board approved this study. All admissions to UIH for COVID-19 positive patients were reviewed for the time of the first COVID-19 positive test and the date of admission. If the first positive COVID-19 test was performed greater than 14 days prior to admission or greater than 48 h after admission, the patient was excluded. Patients transferred from another institution were reviewed for prior COVID-19 testing. If the COVID-19 test was greater than 14 days before transfer, the patient was excluded. If the transfer was not related to any possible COVID-19 symptoms, the patient was excluded. If the patient was discharged and then readmitted less than 14 days after the first positive COVID-19 test, the encounter was included. Patients were discharged or expired prior to 8/18/20. Pregnant patients were included.

Since our goal was to assess the predictive power of our own prediction model as well as some of those in the literature, we partitioned our data into a training cohort consisting of the first 60% of patients admitted prior to 5/9/20 and a test cohort consisting of patients admitted and discharged from 5/9/20 through 8/18/20.

Variable selection was based on a review of the extant literature and expert opinion. The variables selected are shown in Table 1. Admission vital signs, laboratory values and clinical and radiological features were assessed. The results were the first available up to 24 h after admission. Two outcomes were evaluated, mortality (death during hospitalization), and “criticality”, defined as mortality or admission to an ICU.

**Table 1:** Characteristics of the development and test cohorts

Characteristics	Development cohort (N = 309)			Test cohort (N = 207)			P
	Missing Data			Missing Data			
*Outcome variables(N, (%))*							
Mortality	38	(12.3)		21	(10.1)		0.45
Criticality	80	(25.9)		46	(22.2)		0.34
*Demographics*							
Age (Mean, (SD))	56.5	(16.0)		53.3	(18.5)		0.008*
Female (%)	49.8			48.3			0.73
*Race (N (%))*			1%			1.4%	0.22
African American	152	(49.2)		86	(41.5)		
Hispanic	37	(12)		25	(12.1)		
Other, Non- Hispanic	94	(30.4)		82	(39.6)		
White	23	(7.4)		11	(5.3)		
*Vital signs on admission (mean (SD))*							
Systolic blood pressure	135	(25)		134	(24.7)		0.80
Diastolic blood pressure	78.3	(15)		77.9	(14.3)		0.92
Hearth rate	102	(21)		97.1	(20.1)		0.70
Respiratory rate	23.6	(6.9)		22.7	(6.6)		0.52
Temperature	37.5	(1.1)		37.2	(1.0)		0.095
Oxygen saturation	93.4	(7.5)		97.7	(62.4)		0.16
*Clinical and radiological features*							
BMI, mean (SD)	32.3	(10.5)		32.0	(9.6)		0.56
GCS, mean (SD)	14.9	(0.8)	0%	14.8	(1.2)	1%	0.28
Dyspnea (N (%))	125	(40.5)		90	(43.5)		0.49
Coma (N (%))	3	(1)		1	(0.5)		0.54
Pregnant (N (%))	10	(3.2)		14	(6.8)		0.062
Abnormal chest X-ray (N (%))	228	(75)	1.9%	136	(74)	10.6%**	0.67
*Laboratory findings (mean, (SD)*							
White blood cells	6.8	(3.1)	0%	7.7	(3.9)	1%	0.001*
Neutrophiles	5.2	(3.6)	0%	5.6	(3.7)	3.4%	0.038*
Lymphocytes	1.1	(0.7)	0%	1.3	(1)	3.4%	0.024*
Hemoglobin	13.0	(2.2)	0%	12.7	(2.3)	1%	0.57
Hematocrit	39.4	(6.3)	0%	37.9	(6.8)	1%	0.36
RDW	14.9	(2)	0%	15.1	(2.2)	1%	0.68
Platelets	215	(91)	0%	236	(105)	1%	0.15
Creatinine	1.8	(3)	0%	1.9	(2.6)	2.4%	0.47
Lactic acid	1.6	(1.6)	23.3%	1.8	(1.9)	30.4%	0.11
Lactate dehydrogenase	353	(227)	16.5%	386	(521)	27.5%**	0.14
Pro-calcitonin	1.4	(6.7)	16.8%	2.2	(10.4)	32.9%**	0.12
Troponin I	0.11	(0.8)	32.4%	0.04	(0.1)	31.4%	0.076
B-type natriuretic peptide	527	(1939)	57%	369	(664)	63.8%	0.10
Albumin	3.7	(0.5)	3.9%	3.6	(0.6)	7.2%	0.009*
ALT	38.8	(44.4)	3.9%	37.8	(49.9)	7.2%	0.88
AST	48.2	(58.3)	3.9%	52.3	(81.3)	7.2%	0.20
Total bilirubin	0.7	(0.9)	3.9%	0.8	(0.7)	7.2%	0.76
Direct bilirubin	0.2	(0.5)	3.9%	0.2	(0.3)	7.2%	0.57
Creatine kinase	281	(471)	63.8%	3071^**+**^	(22,782)	67.6%	0.012*
C-reactive protein	101	(84)	15.5%	98.6	(86.7)	19.8%	0.46
Interleukin 6	24.9	(33.9)	75.1%	28.1	(40.2)	87.9%**	0.17
D-dimer	1.9	(2.6)	40.8%	2.2	(3)	27.5%**	0.46
Ferritin	884	(1562)	7.1%	930	(1360)	17.4%**	0.45
*Medical condition*							
Hypertension	178	(57.6)		111	(53.6)		0.37
Heart disease	94	(30)		56	(27)		0.41
Stroke	23	(7.4)		11	(5.3)		0.34
Diabetes	161	(52.1)		96	(46)		0.20
Asthma	65	(21)		45	(22)		0.85
COPD	24	(7.8)		13	(6.3)		0.52
Chronic kidney disease	52	(17)		38	(18)		0.65
End-stage renal disease	30	(9.7)		24	(12)		0.49
Cancer	33	(11)		23	(11)		0.88
Transplant	1	(0.3)		1	(0.5)		0.78
Human immunodeficiency virus	7	(2.3)		1	(0.5)		0.11
Immunosuppression	2	(0.6)		9	(4.3)		0.004*
Sickle cell disease	6	(2)		6	(2.9)		0.48
Nicotine use	34	(11)		32	(16)		0.14
Alcohol use	59	(19)		45	(22)		0.46
Substance use	16	(5)		11	(5.3)		0.95
*Variables needed for external models only*							
Blood urea nitrogen, mean (SD)				23.5	(20.7)	1.9%	
eGFR, mean (SD)				70.9	(40.6)	2.4%	
Partial thromboplastin time, mean (SD)				34.4	(6.5)	42.5%	

ALT, alanine aminotransferase; AST, aspartate aminotransferase; BMI, body mass index; COPD, chronic obstructive pulmonary disease; eGFR, estimated glomerular filtration rate; GCS, Glasgow coma scale; RDW, red blood cell distribution width; SD, standard deviation

^*^Continuous variables were compared using a t-test and categorical variables, including missingness, were compared using a Chi-square test

^**^P < 0.05 using a chi-square test, development versus test cohort

Significance was set at 0.05

^**+**^A single very high, but clinically consistent creatine kinase accounted for the very large mean in this group

### Literature search

We searched for articles published in PubMed, Embase, Arxiv and medRxiv using the search string: [Prediction] AND [Human] AND [COVID-19] OR [SARS-COV2] AND [Clinical Trial] OR [Observational Trial] which were published before 8/27/2020. Articles were reviewed to determine whether the models described predicted our outcomes of interest and whether there was sufficient concordance and detail provided to implement the model using our cohort’s data.

### Model development

The objective of our model development was to accurately predict patient outcomes using a reduced number of key input features. A variety of popular machine learning algorithms were evaluated to classify mortality and criticality. These algorithms include Linear Regression [8], Decision Tree [9], Random Forest [10], XGBoost [11], LightGBM [12], and CatBoost [13]. The training process uses a combination of step forward feature selection and parametric grid search. Step forward feature selection is the process of starting with a single feature and iteratively adding one additional feature until there is no increase in model performance. For each step in the feature selection, a parametric grid search is performed to determine the optimal parameter set for each model. We use the area under the receiver operating characteristic curve (AUC) as the evaluation metric.

### Statistical analysis of models

No missing data were imputed in our test cohort. External models were included in our analyses if predictions could be generated for greater than 60% of the patients based on this missingness. If odds or a point scale was available, a receiver operator curve was developed and the area under the curve (AUC) was calculated.

Confidence interval and comparison of ROCs were performed using DeLong’s method [14]. The training and test cohorts were compared using Chi-Square tests for categorical variables and two-sided t-tests for continuous variables using a significance level of P < 0.05. The fraction of missingness for each variable was compared between the cohorts using the Bonferroni correction to control the family-wise error rate.

Descriptive statistics were performed using Stata 12 SE version (StataCorp, TX). Model development was conducted using the Python libraries sklearn (v.0.23.1), TensorFlow (v.2.2.0), XGBoost (v0.90), LightGBM (v.2.3.1) and Catboost (v.0.23.1). Statistical analysis was performed using the pROC package in R. This study was approved by the UIC Institutional Review Board.

## Results

### UIH cohort characteristics model compilation

A description of the UIH cohorts is shown in Table 1. There was a total of 516 patients. The training cohort included the first 309 patients (60%), and the test cohort was the subsequent 207 patients (40%). The test cohort was slightly younger, 53.3 vs 56.5 years [P = 0.008]. Though the whole racial distribution was not significantly different between the cohorts, the proportion of self-declared black patients was 49% in the training cohort and 42% in the test cohort. The lymphocyte, white blood cell and neutrophil counts were significantly higher in the test cohort.

Though some lab tests were performed on almost all patients, many tests were performed in a more discretionary fashion. The missingness of some of the more discretionary tests was higher in the test cohort than in the training cohort: ferritin 7.1–17.4%, Lactate Dehydrogenase (LDH) 16.5–27.5%, Procalcitonin 16.8–32.9%, Interleukin 6 (IL-6) 75.1–87.9%. D-dimer was missing less frequently in the test cohort, 40.8–27.5%.

### Model compilation summary

Ninety-one abstracts were reviewed. After applying our inclusion criteria, 41 articles remained. The models and references are shown in Table 2.

**Table 2:** Prediction models of inpatients matching outcomes of interest

#	Title	Location	Population timepoint/inclusion criteria	N	Outcome	Method^a^	Features	Performance^b^
1	ADL-dependency, D-Dimers, LDH and absence of anticoagulation are independently associated with one-month mortality in older inpatients with Covid-19 [15]	France	Inpatients, > 65-year-old	108	Mortality	Cox regression	ADL-dependency, D-Dimer, LDH, Anticoagulation	AUC 0.83
2	Association of cardiac biomarkers and comorbidities with increased mortality, severity, and cardiac injury in COVID-19 patients: a meta-regression and decision tree analysis [16]	Brazil, China, UK, USA	Inpatients	17,364	Mortality, ICU, mixed	Meta-analysis, decision tree	Age, Troponin I, AST	Precision 74% recall 86%
3	Prediction for progression risk in patients with COVID-19 pneumonia: the CALL Score [17]	China	Inpatients, excluding non-COVID-19 primary infection	248	Deterioration or worsening of CT lung	Cox regression, nomogram	Age, ALC, comorbidities LDH	AUC 0.91
4	Clinical and laboratory predictors of in-hospital mortality in patients with COVID-19: a cohort study in Wuhan, China [18]	China	Inpatients excluding pregnancy & multiorgan failure	296	Mortality	Logistic regression	Age, ALC, SAT, ANC,CRP, D-Dimer, AST, eGFR	AUC 0.88
5	Development and validation of a clinical risk score to predict the occurrence of critical illness of hospitalized patients with COVID-19 [19]	China	Inpatients	710	Criticality	Logistic regression	Age, CXR, Cancer, LDH, Hemoptysis, Dyspnea, Unconsciousness, NLR, comorbidities, Direct bilirubin	AUC 0.88
6	Development and validation of prognosis model of mortality risk in patients with COVID-19 [20]	China	Inpatients	305	Mortality	Logistic regression	Age, CRP, LDH	AUC 0.95
7	Laboratory predictors of death from coronavirus disease 2019 (COVID-19) in the area of Valcamonica, Italy [21]	Italy	Inpatients	144	Mortality	Logistic regression	Age, LDH, CRP, WBC, ANC, ALC, albumin, PTT	80% of variance
8	Prediction model and risk scores of ICU admission and mortality in COVID-19 [22]	USA, New York	Inpatients	641	Mortality	Logistic regression	LDH, PC, smoking, SAT, ALC	AUC 0.82
9	Risk factors associated with clinical outcomes in 323 COVID-19 hospitalized patients in Wuhan, China [23]	China	Inpatients	323	Mortality	Logistic regression	Age, smoking, ALC, ANC, critical disease, DM, Troponin I	Not Reported
10	Risk factors of fatal outcome in hospitalized subjects with coronavirus disease 2019 from a nationwide analysis in China [24]	China	Inpatients	1,590	Mortality	Cox regression	Age, CHD, dyspnea, CVA, PC, AST	AUC 0.91
11	Development of a clinical decision support system for severity risk prediction and triage of COVID-19 patients at hospital admission: an international multicenter study [25]	China, Italy & Belgium	Inpatients, without other severe illness	725	"Severe disease"	Logistic regression	Age, ALC/WBC, CRP, LDH, CK, urea, calcium	AUC 0.88
12	Diagnostic performance of initial blood urea nitrogen combined with D-dimer levels for predicting in-hospital mortality in COVID-19 patients [26]	China	Inpatients	305	Mortality	Cox regression	D-Dimer, BUN	AUC 0.94
13	IL-6-based mortality risk model for hospitalized patients with COVID-19 [27]	Spain	Inpatients	501	Mortality	Logistic regression	ALT, IL6, CRP, LDH, ferritin, ANC, NLR, ALC, albumin, platelets, monocytes, SAT/FiO_2_ ratio	AUC: 0.87
14	Laboratory findings and a combined multifactorial approach to predict death in critically ill patients with COVID-19: a retrospective study [28]	China	Inpatients, severe	336	Mortality	Logistic regression	D-Dimer, ALC/WBC, BUN	AUC 0.99
15	Predictive values of blood Urea nitrogen/creatinine ratio and other routine blood parameters on disease severity and survival of COVID-19 patients [29]	Turkey	Inpatients	139	Mortality	Cox hazard regression	BUN/Cr, NLR	AUC 0.95
16	Prognostic modelling of COVID-19 using artificial intelligence in a UK population [30]	UK	Inpatients	398	Mortality	Artificial neural network	Age, altered mentation, HTN, CLD, collapse, Sex, cough, fever, CKD, DM, CHD, CVA, myalgia, smoking, symptom onset, BMI, diarrhea, vomiting, anosmia, ageusia, cirrhosis, abdominal pain	AUC 0.90
17	Redefining cardiac biomarkers in predicting mortality of inpatients with COVID-19 [31]	China	Inpatients	3219	Mortality	Mixed effects cox model	Troponin I, CK-MB, BNP, CK, Myoglobin	AUC 0.83
18	Risk factors for severe illness in hospitalized Covid-19 patients at a regional hospital [32]	USA, Maryland	Inpatients	117	Criticality	Logistic regression	Oxygen requirement, Sputum production, DM, CKD	AUC 0.88
19	Scoring systems for predicting mortality for severe patients with COVID-19 [33]	China	Inpatients	452	Mortality	Lasso/regression	Age, CHD, D-Dimer, PC, ALC	AUC 0.94
20	Simple nomogram based on initial laboratory data for predicting the probability of ICU transfer of COVID-19 patients: Multicenter retrospective study [34]	China	Inpatients	461	ICU	Cox regression	Age, HTN, ANC, PC, PT, D-Dimer, ALC, albumin	AUC 0.85
21	Clinical characteristics, associated factors, and predicting COVID-19 mortality risk: a retrospective study in Wuhan, China [35]	China	Inpatients	1633	Mortality	Logistic regression	Age, Sex, DM, ALC, PC	AUC 0.76
22	Combination of four clinical indicators predicts the severe/critical symptom of patients infected COVID-19 [36]	China	Inpatients	336	Mortality	Cox regression	Age, GSH, CD3 ratio, total protein	AUC 0.98
23	Identification and validation of a novel clinical signature to predict the prognosis in confirmed COVID-19 patients [37]	China	Inpatients	270	Mortality	Cox regression	Age, CRP, ALC, ANC, PC	AUC 0.95
24	Myocardial injury determination improves risk stratification and predicts mortality in COVID-19 patients [38]	Spain	Inpatients excluding cardiac primary	707	Mortality	Cox regression	Age, sex, CRP, myocardial injury, HTN, RAAS inhibitor, hematocrit, Cr, D-Dimer, CCI	AUC 0.79
25	Neutrophil-to-lymphocyte ratio and outcomes in Louisiana Covid-19 patients [39]	USA, Louisiana	Inpatients	125	Mortality	Cox Regression	NLR (day 2), NLR (day 5)	AUC 0.78
26	Prediction of the severity of Corona Virus Disease 2019 and its adverse clinical outcomes [40]	China	Inpatients	88	Mortality	Logistic regression	Age, ALC, IL 6	AUC 0.97
27	A clinical risk score to identify patients with COVID-19 at high risk of critical care admission or death: An observational cohort study [41]	UK	Inpatients	1157	Mortality and ICU	Lasso/regression	Age, sex, Cr, CKD, CLD, Ethnicity, "index of multiple deprivation", O2 requirement, SAT, respiratory rate, CXR, ALC, ANC, CRP, albumin, cancer, DM, HTN, CHD	AUC 0.76
28	An interpretable mortality prediction model for COVID-19 patients [42]	China	Inpatients	375	Mortality	Decision tree	LDH, CRP, ALC/WBC	AUC 0.99
29	Clinical prediction model for mortality of adult Diabetes inpatients with COVID-19 in Wuhan, China: a retrospective pilot study [43]	China	Inpatients with diabetes	78	Mortality	Logistic regression	PTT, BUN, WBC, LDH	AUC 0.84
30	Development and external validation of a prognostic multivariable model on admission for hospitalized patients with COVID-19 [44]	China	Inpatients	299	Mortality	Logistic regression	Age, LDH, ALC, SAT	AUC 0.98
31	Development and validation of a risk factor-based system to predict short-term survival in adult hospitalized patients with COVID-19: a multicenter, retrospective, cohort study [45]	China	Inpatients admitted w/o other severe illness	828	Mortality	Cox regression	Age, LDH, NLR, Direct bilirubin	AUC 0.88
32	Early prediction of mortality risk among severe COVID-19 patients using machine learning [46]	China	Inpatients	183	Mortality	Logistic regression	Age, CRP, D Dimer, ALC	AUC 0.88
33	Estimation of risk factors for COVID-19 mortality—preliminary results [47]	China	Unclear	N/A	Mortality	Logistic regression	Age, CHD, CLD, Sex	Not reported
34	Evaluation and Improvement of the National Early Warning Score (NEWS2) for COVID-19: a multi-hospital study [48]	UK	Inpatients	439	Criticality	Lasso/regression	Age, BUN, SAT, CRP, eGFR, ANC, NLR, NEWS2, O2 requirement	AUC 0.74
35	Host susceptibility to severe COVID-19 and establishment of a host risk score: findings of 487 cases outside Wuhan [49]	China	Inpatients	487	Mortality and "severe cases"	Logistic regression	Age, sex, HTN	Not reported
36	Prognostic factors for COVID-19 pneumonia progression to severe symptom based on the earlier clinical features: a retrospective analysis [50]	China	Inpatients	125	"Severe" pneumonia	Logistic regression	Underlying disease, respiratory rate, CRP, LDH	AUC 0.98
37	Risk prediction for poor outcome and death in hospital in-patients with COVID19: derivation in Wuhan, China and external validation in London, UK [51]	China	Inpatients	775	Mortality	Logistic regression	Age, sex, ALC, ANC, platelets, CRP, Cr	AUC 0.91
38	Predicting severe COVID-19 at presentation, introducing the COVID Severity Score [52]	Netherlands	Inpatients	261	Respiratory failure	Logistic regression	Age, CRP, ALC, NLR, BUN, LDH, RDW, SAT	AUC 0.79
39	Comorbidity and prognostic factors on admission of a covid-19 cohort in a general hospital [53]	Spain	Inpatients	96	Mortality	Regression	Age, LDH, Cardiomyopathy	Not reported
40	Epidemiology, risk factors and clinical course of SARS-CoV-2 infected patients in a Swiss university hospital: an observational retrospective study [54]	Switzerland	Inpatients	200	Ventilation	Regression	Sex, qSOFA score, CXR, CRP	Not reported
41	Comparison of deep learning with regression analysis in creating predictive models for SARS-CoV-2 outcomes [55]	UK	Inpatients	398	Mortality	Artificial neural network	Confusion, collapse, dyspnea, cough, CKD, heart failure, CVA, fever, sex, CHD, HTN	AUC 0.93

ADL, activities of daily living; ALC, absolute lymphocyte count; ANC, absolute neutrophil count; ALT, alanine aminotransferase; AST, aspartate transaminase; BUN, blood urea nitrogen; CCI, Charlson comorbidity index; CD, cluster of differentiation; CHD, coronary heart disease; CK, creatinine kinase; CK-MB, creatinine kinase-MB; CKD, chronic kidney disease; Cr, creatinine; CRP, C reactive peptide; CLD, chronic lung disease; CXR, chest Xray; CVA, cerebrovascular accident; DM, diabetes mellitus; eGFR, estimated glomerular filtration rate; FiO2, fraction of inspired oxygen; GSH, glutathione reductase; HTN, hypertension; IL,6 interleukin 6; LDH, lactate dehydrogenase; NEWS2, national early warning score 2; [48] NLR, neutrophil to lymphocyte ratio; O2, oxygen; PC, procalcitonin; PT, prothrombin time; PTT, partial thromboplastin time; qSOFA, quick sequential organ failure assessment; RAAS, renin–angiotensin–aldosterone system; RDW, red blood cell distribution width; SAT, oxygen saturation; WBC, white blood cell count

^a^Many studies used multiple methods to help select variables or as trials. The method listed corresponds to the method used to produce the final performance listed

^b^When available the area under the curve (AUC) or c-statistic is shown, if multiple, best is shown. Although other measures may have been performed, they are not shown

^c^ “Criticality” is mortality or ICU stay or ventilation

Over 60% of the models (n = 26) were derived in China, 11 in Europe, 3 in the US and 2 were multinational. The most common methods were logistic regression (n = 25) and Cox Regression (n = 12).A small number of models used neural networks and decision trees. Among models which published an AUC, the AUC’s ranged from 0.74 to 0.98.

### UI health internal model development

Multiple methods of machine learning were assessed to develop the best prediction model of the training (60%) cohort. The best models for both mortality and criticality were random forest models, based on the AUC values. Table 3 lists the key modeling parameters and covariates for the mortality and criticality models. The covariates are listed in the order of importance generated by the step forward regression. The key parameters for the random forest models were determined during the grid search of the development data set. The AUC for the mortality model in the training cohort was 0.98, and for criticality it was 0.97.

**Table 3:** Internal Model Fit on first 60% of admissions for mortality and criticality

Model	Method	Key parameter	Covariates
Mortality	Random forest	Number of estimators: 100 Max depth: 5 Minimum sample Split: 3	Age, diastolic pressure, O2 Sat, BMI, AST, creatinine, CRP, ferritin, platelet, RDW, WBC
Criticality	Random forest	Number of estimators: 100 Max depth: 5 Minimum sample Split: 2	Age, O2 Sat, ALT, AST, creatinine, CRP, ferritin, platelet, RDW, WBC, neutrophil/lymphocyte ratio

ALT, alanine aminotransferase; AST, aspartate aminotransferase; BMI, body mass index; CRP, C-reactive protein; O2 Sat, oxygen saturation; RDW, red blood cell distribution width; WBC, white blood cell count

If model coefficients in the papers in Table 2 were sufficiently described and the model variables were available for more than 60% of admissions, the model was used to predict outcomes in the UIH test cohort. Results are shown in Table 4.

**Table 4:** Prediction models fit to UIH test cohort

Study	Cohort origin	N (develop)	Outcome	Method	Covariates	Performance	Test cohort # evaluable	Test cohort performance
A] An interpretable mortality prediction model for COVID-19 patients [42]	Wuhan, China	351	Mortality*	Decision tree	(3) CRP, LDH, lymphocyte percentage	PPV 96.9% NPV 98.4%	145 (70%)^***^	AUC 0.69 (0.60–0.79)
B] Development and validation of a clinical risk score to predict the occurrence of critical illness of hospitalized patients with COVID-19 [19]^a^	China	1590	Criticality**	Logistic regression (LR)	(10) Age, cancer, direct bilirubin, comorbidities, dyspnea, hemoptysis, LDH, neutrophils/lymphocytes, unconscious, CXR	AUC 0.88 (0.84–0.93)	144 (70%)	AUC 0.84 (0.78–0.91)
C] Development and validation of prognosis model of mortality risk in patients with COVID-19 [20]	Wuhan, China	292	Mortality	LR	(3) Age, LDH, CRP	AUC 0.95	141 (69%)	AUC 0.89 (0.82–0.96)
D] Diagnostic performance of initial blood urea nitrogen combined with D-dimer levels for predicting in-hospital mortality in COVID-19 patients [26]	Wuhan, China	305	Mortality	LR	(2) BUN, D-dimer	AUC 0.94 (0.90–0.97)	150 (72%)	AUC 0.73 (0.62–0.83)
E] Laboratory findings and a combined multifactorial approach to predict death in critically ill patients with COVID-19: a retrospective study [28]	Wuhan, China	336	Mortality	LR	(3) BUN, D-dimer, lymphocyte percentage	AUC 0.99 (0.98–1.0)	148 (71%)^***^	AUC 0.72 (0.61–0.82)
F] Development and external validation of a prognostic multivariable model on admission for hospitalized patients with COVID-19 [44]	Wuhan, China	299	Mortality	LR	(4) Age, LDH, lymphocyte count, O2 Saturation	AUC 0.98 (0.96–1.0)	150 (72%)	AUC 0.84 (0.73–0.94)
G] Early prediction of mortality risk among severe COVID-19 patients using machine learning [46]^b^	Wuhan, China	183	Mortality	LR	(4) Age, CRP, D-dimer, lymphocyte count	AUC 0.90	142 (69%)	AUC 0.68 (0.51–0.81)
H] Risk prediction for poor outcome and death in hospital in-patients with COVID19: derivation in Wuhan, China and external validation in London, UK [(51)]^c^	Wuhan, China	775	Mortality	LR	(7) Age, CRP, sex, Cr, lymphocytes, neutrophils, platelets count	AUC 0.91	165 (80%)	AUC 0.72 (0.58–0.86)
UIH mortality model	Chicago, USA	309	Mortality	Random forest	(11) Age, AST, BMI, Cr, CRP, diastolic BP, ferritin, O2 saturation, platelet count, RDW, WBC	AUC 0.98 (0.96–1.0)	152 (73%)	AUC 0.84 (0.74–0.94)
UIH criticality model	Chicago, USA	309	Criticality	Random forest	(11) Age, ALT, AST, Cr, CRP, ferritin, RDW, neutrophils/lymphocytes, O2 saturation, platelet count, WBC	AUC 0.97 (0.94–1.0)	152 (73%)	AUC 0.83 (0.76–0.90)

ALT, alanine aminotransferase; AST, aspartate transaminase; BMI, body mass index; BP, blood pressure; BUN, blood urea nitrogen; Cr, creatinine; CRP, C reactive peptide; CXR, chest Xray; LDH, lactate dehydrogenase; O2, oxygen; PC, procalcitonin; RDW, red blood cell distribution width; SAT, oxygen saturation; WBC, white blood cell count

^*^Mortality defined as death prior to discharge**Criticality defined as mortality or intensive care unit stay

^***^For these models only non-pregnant patients were used. The other models either included or did not specify inclusion of pregnant patients

^a^http://118.126.104.170/

^b^https://phenomics.fudan.edu.cn/risk_scores/

^c^https://covid.datahelps.life/prediction/

A total of 10 models were assessed using the test cohort, 8 from the literature and 2 internal. Seven of the external models used logistic regression and one used a decision tree. One external model predicted criticality; the remainder predicted mortality. The most common variables used in the models were the age (7 models), lymphocyte count or lymphocytes/WBC ratio (6 models), C-reactive protein (CRP) and LDH (4 models), D-dimer (3 models) and BUN (2 models). The number of features used in each model ranged from 2 to 11, with a median of 3.5. These models assessed clinical features and laboratory testing upon admission. In addition, 1 model explicitly included pregnant patients [19], 2 excluded pregnant patients [28, 42], and 5 were undetermined [20, 26, 44, 46, 51].

Three of the models, B, G and H [19, 46, 51], had open access web-based calculators to predict outcomes for individual patients. One model used a decision tree of only three variables which is easy for a clinician to use (A) [42]. Two models used a nomogram to try to simplify use (D and F) [26, 44].

All external models were trained using cohorts of Chinese patients. Though there were non-Chinese cohort models in Table 2, none of them provided sufficient description of their models to be implemented on our test cohort without retraining.

Common reasons why models were not used were the lack of availability of the coefficients needed to calculate a prediction score, lack of concordance between the features used in the model and features available in our test cohort, and outcome data not available in our test cohort (e.g., mortality).

**Fig. 1:**
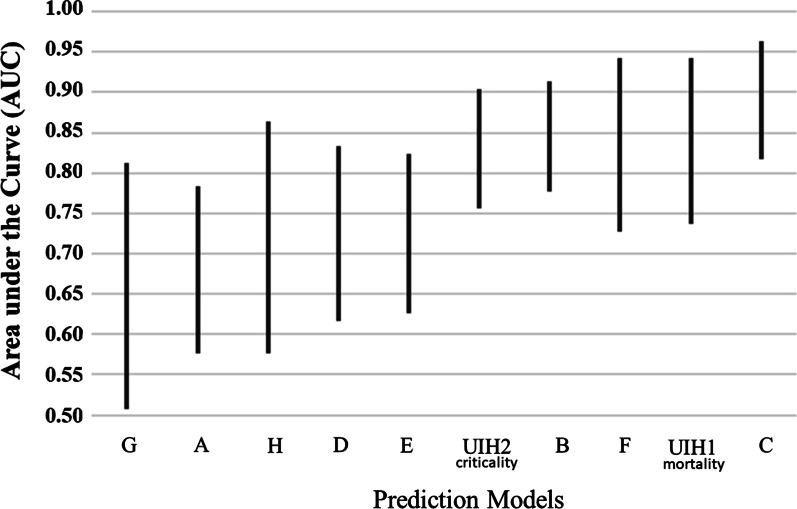
Area under the curve (AUC) confidence intervals for Table 4 models

Figure 1 shows the confidence intervals of the AUC’s obtained on the test cohort. Table 4 and Fig. 1 show that the best estimate for the AUC ranges from 0.68 for model G to 0.89 for model C. The internal models have an AUC of 0.84 for mortality and 0.83 for criticality. The mortality model with the highest AUC, C, was not statistically different than the UIH mortality model, 0.89 (0.82–0.96) vs AUC 0.84 (0.74–0.94), [P > 0.5].

The confidence intervals range from 0.13 to 0.30. The difference in performance between the published fit and that of its performance on our test set varied significantly. For model B this difference in AUC was only 0.04 and for model E it was 0.26. The UI Health models were in the middle with a 0.14 AUC difference.

For all 8 models, the mean values for lab results and those of the UI Health test cohort are shown in Table 5. The variables shown were used in at least one model and were available in five or more of the model cohorts. Age and CRP were reported in all papers. The creatinine was reported in seven papers. Though rigorous statistical testing cannot be performed due to the inability to obtain the raw data, some of the variables are clinically significantly different between the cohorts from China and UIH. The mean CRP at UIH is more than three times higher than in the external model average, the creatinine is two-fold higher and the LDH is roughly 1/3 higher.

**Table 5:** Values of the most common variables in the 8 external models and the test cohort

Characteristics	Age (N = 8)	CRP (mg/L) (N = 8)	Cr (mg/dl) (N = 7)	LDH (U/L) (N = 6)	Lymph (1000/uL) (N = 6)	Lymph/WBC (N = 6)	Neut (1000/uL) (N = 4)
*Model cohort*							
A	58.8	26.3*	N/A	***274***	***N/A***	***0.14***	***N/A***
B	***48.9***	34.8	0.86	***314***	***1.4***	***N/A***	***4.1***
C	***56.5***	***21.1***	1.08	***148***	***N/A***	***0.24***	***N/A***
D	65.0	22.5	0.78	272	1.1	0.19	4
E	65.0	6.2	0.76	N/A	1.3	0.23	3.9
F	***62.0***	64.5	0.83	***362***	***0.8***	***0.11***	***N/A***
G	***63.5***	***41.3***	0.84	345	0.9	0.14	N/A
H	***61.0***	***2.7***	***0.72***	N/A	1.4	N/A	3.5
Total (Mean, (SD))	60.1 (5.4)	27.4 (19.8)	0.84 (0.12)	286 (77)	1.1 (0.3)	18% (5)	3.9 (0.3)
UIH cohort**							
Mean (SD)	***53.3*** (18.5)	***98.6*** (86.7)	***1.93*** (2.63)	386 (521)	1.3 (1)	***18%*** (11)	5.8 (3.6)
Median (IQR)	55 (40–67)	75.2 (32–146)	1.02 (0.8–1.6)	297 (230–417)	1.1 (0.7–1.5)	16% (10–24)	4.7 (3.3–7.3)

^*^ Bolded, italicized, underlined values represent variables used in the final models

^**^These values are for the entire cohort, validate and test, N = 516

* Cr* creatinine, *CRP* C reactive peptide, *IQR* interquartile range, *LDH* lactate dehydrogenase, *Neut* neutrophile count, *Lymph* lymphocyte count, *Lymph/WBC* lymphocyte to white blood cell ratio, *SD* standard deviation, *UIH* University of Illinois Hospital

## Discussion

All the models in Table 1 could not be used to make predictions on our test cohort for multiple reasons. Without chart review, symptomology and its duration are difficult to obtain, excluding some models. Unusual imaging grading schemes or mandatory CT scans were not available in our cohort. Some studies used labs that were not ordered frequently in our hospital. Lack of longitudinal follow up limited the use of timed mortality, i.e., 30-day etc. These issues, along with the lack of well described coefficients of models produced the inability to use models except for the 8 models in Table 1.

The features used in the models were surprisingly diverse. The number of variables in each model ranged from 2 through 11, with 19 different variables across the studies. The most common variables used were age, lymphocyte count, CRP and LDH. It is surprising that only 7 of the 10 models used age as a predicting variable, and the 3 models that did not use it did not perform well. In large multi-site cohorts examined in Britain [56], the US [57] and internationally [58], age was a strong predictor of mortality.

Three of the external models performed very well, with AUC’s of 0.84–0.89. This demonstrates that although the patients were geographically distant, ethnically different, in different health systems and cultures, and at different times during the pandemic, reasonable prediction was possible. Our initial hypothesis was that these models would not work well, but this was not the case in all the models.

It is likely that some of the models may have had better performance if retrained using our local cohort, but this was not done as the purpose was to see how they worked “out of the box”. This appeared to be the intent of many of the authors of the published models as evidenced by the publishing of web calculators, nomograms and decision trees. One of the issues which may cause worse or better performance in a model is that the outcomes have been found to be a function of time during the pandemic, not just patient factors, with improving outcomes more recently [59, 60].

Models A, F, G and H were also evaluated in a review and cohort prediction comparison by Gupta et al. [61] using their cohort of 440 patients from London with a mortality rate of around 28%. For Models F, G and H, the AUCs in our cohort were slightly different than in the London Cohort [61] respectively, model F, 0.84 vs. 0.76, model G, 0.68 vs. 0.74 and model H, 0.72 vs. 0.69.

Review of the characteristics of the cohorts in Table 5 is instructive in understanding why some of the models did not perform well. Model A is a decision tree based on only 3 features, CRP, LDH and the percentage of lymphocytes. The first decision node suggests mortality if the LDH is greater than 365 U/L. In their cohort, the average LDH was 274 U/L. The average LDH in our test cohort was 386 however, thus a large portion were predicted to die at the first node, causing a poor positive predictive value (PPV). In the London cohort the average LDH was about the same as ours, 395 U/L, and this model performed poorly in that cohort also [61].

The average LDH was roughly 1/3 higher in our test cohort than in the average of the cohorts from China. It is not clear what the reason for this is. In a healthy multiethnic cohort from Hawaii [62], there were at most minor differences between black, Hispanic, White and Asian patients in their LDH, suggesting that the differences in LDH are not likely due to racial factors. It is possible that a difference in the time of infection to presentation might explain the difference. The other models which used LDH predicted well, but this might be in part related to use of a logistic regression instead of a decision tree.

The average CRP in our cohort is roughly 350% of the average in the external models, 99 mg/L vs. 27 mg/L. Four models used the CRP and only one model performed well, model C. The creatinine was significantly higher in our cohort than in any of the derivation cohorts and as well as the average of the studies, 0.84 mg/dL. Only one model used the creatinine, model H. Its derivation cohort average creatinine was 0.72 mg/dL. Thus, model H used both the CRP and creatinine, helping explain its poor performance. For creatinine, there are studies showing socioeconomic and ethnic variations in chronic kidney disease [63] with one systematic review showing the prevalence of chronic kidney disease in China was less than a fourth of the rate in the US [64]. The higher creatinine in the test cohort may not be related only to differences in illness at presentation, but rather differences in the prevalence of CKD.

It is not fully clear why the models produced at UIH using our training cohort did not perform better on our test cohort, though there are some likely factors. The AUC for mortality decreased from 0.98 to 0.84 and for criticality, from 0.97 to 0.83. In analysis of the entire cohort, we were able to determine that the mortality and criticality were associated with the admission date. This is consistent with publications showing an improved mortality rate over time [59, 60]. The WBC, lymphocytes and neutrophils were not all used in each model and all went up in the test cohort. Thus, it is possible that some variable not in the models changed over time, producing a worse fit compared to the first 60% of patients.

The number of cases for which a model is unable to generate a prediction due to missing data is an important practical consideration for model implementation. The fraction of the test cohort for which predictions could not be generated due to missingness ranged between 17 and 31% for external models. The UI Health models could not generate predictions in 27% of the patients. Though retrospectively missing data can be imputed, this is not so easy in real time by clinicians during patient care, so was not done. This demonstrates non standardized test ordering, which is not surprising as our understanding of what is useful and necessary for testing in suspected COVID patients has evolved.

It is interesting to note that many of the tests which have been used commonly in these and other models were missed more frequently in the test cohort than the earlier development cohort. Ferritin 17.4% from 7.1%, LDH 27.5% from 16.5%. It is not clear why these tests were ordered less over time, particularly LDH with many publications demonstrated its prognostic power [15, 16, 21, 22, 25, 27, 42–45, 50, 52, 53]. It is possible that the ordering of these inflammatory prognostic markers [65] decreased as clinicians’ confidence with clinical prognosis improved.

D-dimer on the other hand was missing less frequently in the test cohort, 27.5% from 40.8%. This difference may be due to an increased concern for venous thromboembolism in COVID 19 infections [66] which developed over time.

An important question is what model to use to provide prognostic information to clinicians. Using your own data to inform future care is consistent with a learning health system [67]. The ideal situation is that clinical decision support (CDS) could supply the best prediction for a patient based on the most recent trends at the time. Another reason to use your own data, especially with COVID-19, is that the disease, treatment and outcomes are likely to change over time [59, 60], while the models in the literature are static. An additional benefit of using your own data and predictive models is the ability to see which diagnostic tests are most useful prognostically, but are not ordered enough, leading to more evidenced based order sets.

Our literature search has limitations due to the inability to ensure that all possible synonyms were used along with other reasons that the search strategy may have missed articles. As related to COVID-19, the rate of discovery and publication is so rapid that many models were likely published between the time of study completion and study publication.

Limitations related to our cohort and analysis are first that this is a single site study, and these models may have performed differently at other sites. The size of the test cohort contributed to the relatively large confidence intervals of the AUC’s, making statistical significance difficult to prove. We were unable to follow patients consistently after discharge, thus could not measure timed outcomes like 30-day mortality. Lastly, we could not control for changes in treatment which have occurred over time.

## Conclusions

Both internal and some external models were found to work well at predicting mortality in our test cohort. The 3 best external models used at least age, LDH and lymphocytes. Inconsistent ordering of lab tests led to the inability to generate predictions for 27–31% of our cohort using the 3 best external models and the 2 UIH models.

As not all the external models worked well, it would be difficult to know which model to use for future admissions at a particular time during the pandemic as treatment and patient mix can change. As an institution’s own prior patients are most similar to their next group of patients, using models from local data should be considered.

## Data Availability

The datasets generated and/or analyzed during the current study are not publicly available due privacy but are available from the corresponding author on reasonable request.

## Abbreviations

ALC: Absolute lymphocyte count
ANC: Absolute neutrophil count
AST: Aspartate transaminase
BUN: Blood urea nitrogen
CHD: Coronary heart disease
CK: Creatinine kinase
CKD: Chronic kidney disease
Cr: Creatinine
CRP: C reactive peptide
CLD: Chronic lung disease
CXR: Chest Xray
DM: Diabetes mellitus
eGFR: Estimated glomerular filtration rate
HTN: Hypertension
IL 6: Interleukin 6
LDH: Lactate dehydrogenase
NEWS2: National Early Warning Score 2
NLR: Neutrophil to lymphocyte ratio
PC: Procalcitonin
PT: Prothrombin time
PTT: Partial thromboplastin time
RDW: Red cell distribution width
SAT: Oxygen saturation
Stan Dev: Standard deviation
UIH: University of Illinois Hospital
WBC: White blood cell count
